# Virtual Reality Headset as a Cause of Allergic Contact Dermatitis in a Child

**DOI:** 10.7759/cureus.94906

**Published:** 2025-10-19

**Authors:** Dalia Eid, Shaheen Akhtar, Natalie Stone

**Affiliations:** 1 Dermatology, Aneurin Bevan University Health Board, Newport, GBR

**Keywords:** allergic contact dermatitis, gaming accessories, paediatric dermatitis, patch testing, virtual reality headset

## Abstract

Allergic contact dermatitis (ACD) from gaming accessories is rarely reported. With the increasing popularity of virtual reality (VR) headsets, more cases may emerge.

We report the case of a 13-year-old boy with atopic eczema who developed a recurrent periorbital rash at sites contacting an Oculus Quest 2 headset (Meta Platforms, Menlo Park, CA, USA). He was otherwise healthy, on no medications, and had a family history of asthma. Patch testing included the British standard, medicament, facial, and photosensitivity series, with the headset foam added on day two. Positive reactions were observed to methylisothiazolinone, methylchloroisothiazolinone/methylisothiazolinone (MCI/MI), 2-bromo-2-nitropropane-1,3-diol, methyldibromoglutaronitrile, benzisothiazolinone, propolis, Carba Mix, and the headset foam. Symptoms resolved fully following replacement of the foam interface with a silicone cover.

This case expands the spectrum of wearable-device-related allergic contact dermatitis and underscores the importance of including device materials in patch-test panels. It also highlights the need to consider wearable electronics as emerging sources of paediatric ACD.

## Introduction

Gaming is widespread in the United Kingdom, with video game users projected to reach 57.6 million by 2027 [1]. Among children aged seven to 18 years, over two-thirds own or have access to a console. Virtual reality (VR) headsets are increasingly popular, and repeated skin contact with these devices may trigger allergic reactions. Although allergic contact dermatitis (ACD) from gaming accessories is infrequently reported, clinicians should remain vigilant for this emerging cause of facial rashes in children.

ACD is a delayed type IV hypersensitivity reaction mediated by antigen-specific T lymphocytes, leading to localized inflammation and eczematous skin changes upon re-exposure to an allergen [2,3]. VR headsets commonly contain foam padding, rubber, polyurethane, and various plastics, which may include sensitizing agents such as formaldehyde, isocyanates, and rubber accelerators. Continuous skin contact, friction, and perspiration during gameplay may facilitate allergen absorption and trigger dermatitis in susceptible users [4-6].

We present the first reported case of VR headset-associated ACD in a paediatric patient, confirmed by patch testing. This work was presented orally at the British Association of Dermatology Annual Conference, July 2024. We report a case of VR headset-associated ACD in a 13-year-old boy.

## Case presentation

A 13-year-old boy with a history of atopic dermatitis, predominantly mild with only minor dryness on the body managed with topical emollients, presented with an eight-year history of recurrent facial eczema. His facial flares had previously been intermittent and mild. He had a family history of asthma, was not on any regular medications, and his hobbies included climbing trees and playing on his Xbox console.

More recently, following use of an Oculus Quest 2 virtual reality headset (Meta Platforms, Menlo Park, CA, USA), the only VR device he had used, he developed a persistent, eczematous rash localized to the periorbital region and upper cheeks, corresponding to areas in direct contact with the headset. Despite regular use of emollients, including Epaderm ointment and Cetraben cream, the rash showed no improvement.

**Figure 1:**
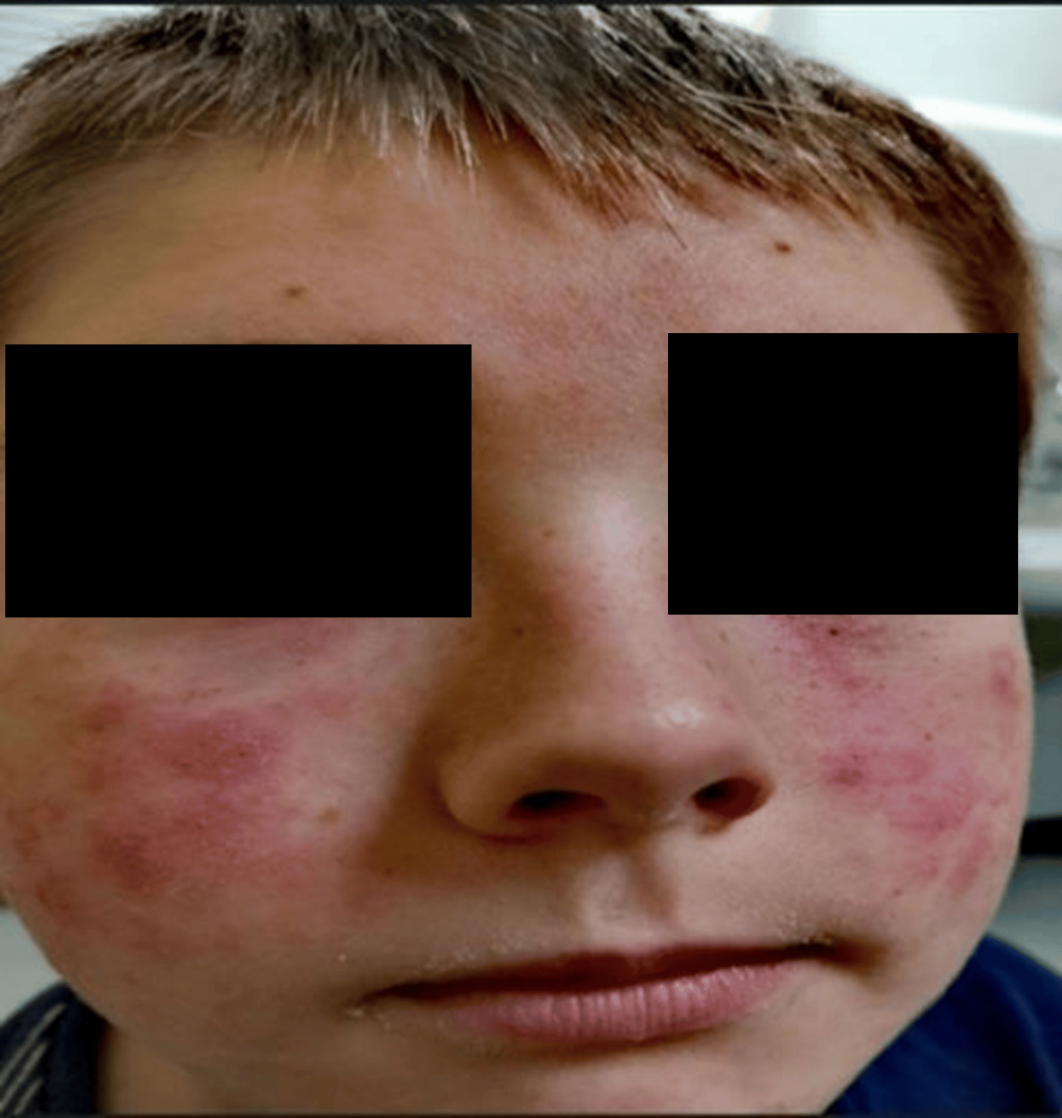
Eczematous rash localized to the cheeks and periorbital areas corresponding to VR headset contact sites

On examination, the eruption was eczematous, with erythema and scaling around the eyelids and extending onto the upper cheeks where the headset foam rested (Figure 1). The chronic visibility of the rash had a marked impact on his confidence and social comfort.

**Figure 2:**
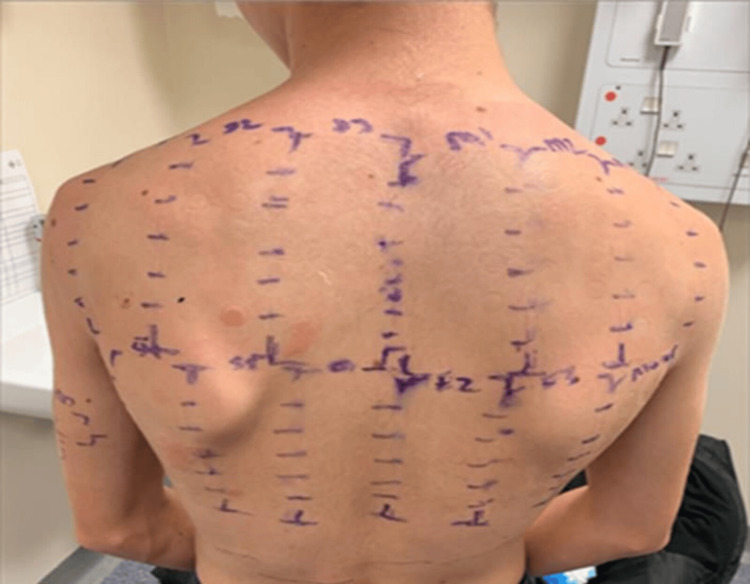
Photo of the back showing positive patch test results of day four

Given the persistent course, characteristic distribution, and suspected contact trigger, patch testing was performed. He underwent testing with the British Standard, medicament, facial, and photosensitivity series, in addition to a sample of the VR headset foam (Chemotechnique Diagnostics, Vellinge, Sweden). Tests were applied in Finn chambers with Hypafix dressings (BSN Medical, Stockholm, Sweden), and readings were taken on days two and four according to International Contact Dermatitis Research Group (ICDRG) criteria (Figure 2). Positive reactions were observed, summarized in Table 1.

**Table 1:** Summary of patch test results This table summarizes the patient’s patch test results, including allergens, concentrations, reactions on days two and four, and clinical relevance.

Allergen	Day 2	Day 4	Relevance
Methylisothiazolinone 0.2%	++	++	Present in shampoo
Methylchloroisothiazolinone/Methylisothiazolinone 0.02% (MCI/MI)	+	+	Present in shampoo
2-Bromo-2-Nitropropane 0.5%	+	+	Cooling spray
Benzisothiazolinone 0.1%	+	+	? Relevance; possibly related to memory foam
Propolis	+	+	Unknown relevance
Carba Mix 3%	+	++	? Relevance; possibly related to VR headset foam
VR headset foam (as is)	+	+	Present in VR headset; causing allergic contact dermatitis (ACD)

The patient strongly suspected the headset foam as the trigger. After consultation with the manufacturer, a medical-grade silicone cover replaced the foam component, resulting in complete resolution of symptoms and allowing continued use of the device without recurrence.

## Discussion

The Oculus Quest 2 headset is composed of memory foam and polyurethane leather; however, the exact chemical composition of the foam is not publicly disclosed. Chemical analysis was not feasible, as it is not routinely performed and presents logistical challenges. In this patient, positive patch test reactions were observed to Carba Mix and benzisothiazolinone, which may have been present in the foam material. Other reactions included 2-Bromo-2-nitropropane (historically used in cooling sprays), methylisothiazolinone/methylchloroisothiazolinone (MI/MCI) present in the patient’s shampoo, and propolis of uncertain relevance. Octylisothiazolinone, previously implicated in headphone-associated ACD [7], tested negative. Isocyanates, known potential allergens in memory foam, were not tested due to limited back-space availability [5].

Although literature on VR headset-induced ACD is limited, allergic reactions to other wearable electronics, including headphones, smartwatches, glucose sensors, and insulin pumps, have been described [7-9]. Barrier dysfunction in atopic individuals likely contributed to the chronic, localized eruption observed in this patient [5,9]. Previous studies have also shown that atopic children may be at higher risk for device-related ACD [8,9]. Winston FK reported wearable device dermatitis in a case of acrylate-related contact allergy [10], and one report documented acute urticaria following VR headset use [6].

Media reports highlighted that Oculus Quest 2 headset pads were recalled due to skin reactions [11,12]. The persistent facial distribution in this patient highlights the importance of recognizing wearable devices as potential sources of chronic ACD in pediatric patients. Barrier measures, such as medical-grade silicone covers, provided a simple and effective management strategy, allowing continued use of the headset without recurrence [9].

Given the expanding use of VR headsets for gaming, education, and occupational purposes, clinicians should maintain a high index of suspicion for device-related ACD in paediatric patients presenting with unexplained facial rashes. Early recognition, targeted patch testing, and practical interventions can prevent prolonged morbidity and reduce psychosocial impact [7-9].

## Conclusions

This case demonstrates that VR headsets can trigger persistent, localized facial dermatitis in children. Identification of the causative allergens through patch testing, combined with practical interventions such as medical-grade silicone covers, can effectively resolve symptoms and permit continued device use. Awareness of wearable device-related contact dermatitis is important to ensure timely diagnosis and management in paediatric patients.
